# Epiphysiodesis for limb length discrepancy: a comparison of two methods

**DOI:** 10.1007/s11751-013-0180-9

**Published:** 2013-11-23

**Authors:** L. V. Babu, O. Evans, A. Sankar, A. G. Davies, S. Jones, J. A. Fernandes

**Affiliations:** Sheffield Children’s Hospital, Western Bank, Sheffield, Yorkshire S10 2TH UK

**Keywords:** Epiphysiodesis, Limb length discrepancy, Canale, Metaizeau

## Abstract

A retrospective review of 42 patients from 1999 to 2008 with at least 1-year follow-up was performed. The type and location of epiphysiodesis, average operative time and hospital stay, complications, timing and the final limb length discrepancy (LLD) were recorded. Computer tomography scanograms and mechanical axis view with grids were done to assess LLD. Twenty-six patients underwent Canale type epiphysiodesis compared with 14 receiving Metaizeau screw epiphysiodesis. The average operation time for Canale type was 42 and 45 min for screw epiphysiodesis. In the Canale group, there was a mean reduction in 2.5 cm in LLD from 3.7 to 1.2 cm over an average follow-up of 2.1 years. There were 4 minor and 2 major complications with a 92 % success rate defined as achieving the desired discrepancy correction. In the screw epiphysiodesis group, the mean change was 1.8 cm from 3.2 to 1.4 cm, over 2.2 years with 2 minor and 2 major complications and a success rate of 85 %. Percutaneous epiphysiodesis by any method is a reliable, minimally invasive method with minimal morbidity and an acceptable complication rate when compared to a corrective osteotomy or an open Phemister-type epiphysiodesis. This study has led to our preference for the Canale method, which in our hands has fewer complications and is more successful at reaching the desired discrepancy correction.

## Background

Epiphysiodesis is a well-established procedure to correct the deformity in those patients with a limb length discrepancy of <5 cm [1–5]. A number of modalities have been described to delay the growth of one limb to allow growth in the contralateral limb and correct the overall leg-length discrepancy [6–8]. Percutaneous epiphysiodesis using the technique of Metaizeau has now widely become the method of choice [9] using screw fixation, but the use of Canale’s technique to ablate the physis by drilling and subsequent burring has also been accepted as a viable option [10]. The use of staples to arrest the growth plate, along with more recent ‘8’ plates, has also been described and may be used.

The aim of the study was to determine the difference in the efficacy of these procedures in treating those patients with moderate limb length discrepancy.

## Methods

A retrospective review of cases was undertaken from 1999 to 2008. Cases were excluded if the leg-length discrepancy was >5 cm; simultaneous lengthening procedures were performed on the contralateral side; follow-up was under 1 year and if the medical notes and radiographs were incomplete. As part of the routine assessment of the patients, clinical evaluation of the leg-length discrepancy was assessed using the block test. Radiological assessment was also measured using computed tomography scanograms and long-leg radiographs to confirm accurate assessment of the discrepancy along with radiographs of the left wrist to accurately determine bone age. Both the Moseley straight-line chart [11] and Paley’s multiplier method [12] were used to estimate the resultant leg-length discrepancy at maturity.

The pre-operative discrepancy was recorded along with the mean duration of follow-up and the discrepancy remaining at final follow-up. The site and time taken to perform each surgery was recorded, as was the duration of hospital stay. Minor and major complications of each surgery were documented.

## Results

A total of 40 suitable cases were identified consisting of 23 males and 17 females with a mean age of 13.3 and 11.8 years, respectively. Moseley charts were used for the 14 cases undergoing the Metaizeau technique, giving an average estimated LLD at maturity of 4.5 cm. In the Canale technique group, 13 cases Paley’s multiplier was used to give a mean estimated LLD of 4.8. The right was found to be involved in 24 cases and the remaining 16 in the left; 20 cases involved the distal femur, 8 involved the proximal tibia and 12 cases involved both tibia and femur. The distribution of operative site was equally comparable between each group. Follow-up was from 12 to 72 months with a mean of 2.2 years. Causes of leg-length discrepancy were found to be diverse with 16 cases from congenital causes including developmental dysplasia of the hip, congenital short femur and proximal focal femoral deficiency. Seven cases were as a result of Perthes disease, 5 were identified as post-traumatic and 3 from previous slipped capital femoral epiphysis. The remaining 9 cases were from other causes.

The Canale method was used in 26 cases and Metaizeau in 14 cases. The mean operative time and hospital stay for each procedure are shown in Table 1.

**Table 1:** Operative time and length of stay

	Canale	Metaizeau
Mean operative time (min)	42	45
Mean hospital stay (day)	1	1

The outcome of leg-length discrepancy for each procedure and mean follow-up for each are shown in Table 2.

**Table 2:** LLD pre-op and at final follow-up with mean follow-up time

	Canale	Metaizeau
Mean pre-operative LLD (cm)	3.7	3.2
Mean predicted LLD at maturity (cm)	4.5	4.8
Mean LLD at final follow-up (cm)	1.2	1.4
Mean duration of follow-up (years)	2.1	2.2

Major complications were defined by the need for further surgery before the end of the follow-up period or failure to arrest growth. Minor complications were defined as complications not requiring further surgery. The number of complications is shown in Table 3 and consisted one failure of arrest and one persistent knee pain requiring screw removal in the Metaizeau technique and two cases of failure to arrest growth in the Canale group. Minor complications included 2 effusions that resolved spontaneously in the Metaizeau group, and in the Canale group, 2 cases developed superficial infections that resolved with antibiotics and 2 cases of haematoma formation, which resolved with non-operative measures.

**Table 3:** Complications for each procedure group

	Canale	Metaizeau
Cases	26	14
Major complication	2	2
Complication rate	7.7 %	14 %

## Discussion

Percutaneous epiphysiodesis is a simple and safe method to correct moderate leg-length discrepancy. There are many different methods of achieving growth arrest, but in this series, the Canale method has been shown to be more effective than percutaneous screw fixation with an increased reduction in mean leg-length discrepancy of 2.5 cm against a mean change of 1.8 cm when using the Metaizeau technique. This was found despite the estimated mean LLD at maturity being greater in the Metaizeau compared to the Canale group and whilst this was not significantly different confirms equivalent remaining growth times in both groups.

Complications were present in both groups of treated patients, but it was found that the rate was half when using a percutaneous epiphysiodesis as opposed to percutaneous screw fixation and at the same time reducing the need for retained metal hardware and potential subsequent surgery to remove problematic metalwork. Whilst the total numbers in each group are not large enough to allow for an adequately powered study, there is an observed difference in the lower rate of complications requiring further surgery in the group treated using the Canale method.

The time taken to perform each surgery was found to be no different, and the hospital stay was not increased when using the Canale method making it a viable and effective means to treat leg-length discrepancy. The need to employ screw fixation in the Metaizeau technique also increases the cost of the procedure with a single 6-mm cannulated screw costing around £99.

We have found that in our unit, the Canale technique offers a suitable, safe, effective and efficient method of treating those children that present with a moderate leg-length discrepancy. We have found that over the last 10 years, this method has reduced morbidity and subsequent hospital stay, gives a good cosmesis, avoids the use of implanted metalwork and achieves a good reduction in the subsequent leg-length discrepancy.
